# Surface Trafficking of APP and BACE in Live Cells

**DOI:** 10.1111/tra.12270

**Published:** 2015-04-14

**Authors:** Anna Bauereiss, Oliver Welzel, Jasmin Jung, Simon Grosse‐Holz, Natalia Lelental, Piotr Lewczuk, Eva M. Wenzel, Johannes Kornhuber, Teja W. Groemer

**Affiliations:** ^1^ Department of Psychiatry and Psychotherapy Friedrich‐Alexander‐Universität Erlangen‐Nürnberg (FAU) Schwabachanlage 6 91054 Erlangen Germany; ^2^ Institute for Cancer Research, Department of Biochemistry The Norwegian Radium Hospital Montebello N‐0310 Oslo Norway

**Keywords:** β‐secretase, APP, BACE, exocytosis, HeLa cells, trafficking pathway

## Abstract

Amyloid‐β (Aβ)‐peptide, the major constituent of the plaques that develop during Alzheimer's disease, is generated via the cleavage of Aβ precursor protein (APP) by β‐site APP‐cleaving enzyme (BACE). Using live‐cell imaging of APP and BACE labeled with pH‐sensitive proteins, we could detect the release events of APP and BACE and their distinct kinetics. We provide kinetic evidence for the cleavage of APP by α‐secretase on the cellular surface after exocytosis. Furthermore, simultaneous dual‐color evanescent field illumination revealed that the two proteins are trafficked to the surface in separate compartments. Perturbing the membrane lipid composition resulted in a reduced frequency of exocytosis and affected BACE more strongly than APP. We propose that surface fusion frequency is a key factor regulating the aggregation of APP and BACE in the same membrane compartment and that this process can be modulated via pharmacological intervention.

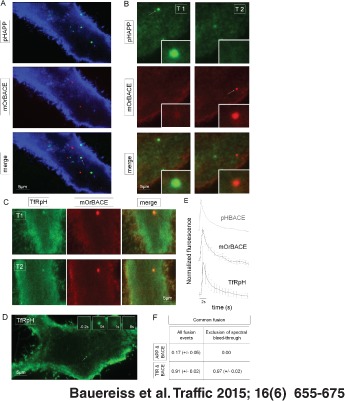

Alzheimer's disease is the most common form of dementia 1. The pathological correlates of this disease include neurodegeneration and the development of characteristic neurofibrillary tangles and senile plaques. These plaques are predominantly composed of amyloid‐β (Aβ) deposits, which are derived from Aβ precursor protein (APP). This extensively studied type 1 transmembrane protein plays physiological roles that are related to dendritic spine and synaptic function 2, 3. Processing of APP to Aβ‐peptide results in the pathology of Alzheimer's disease 4. β‐Site APP‐cleaving enzyme (BACE), an aspartate protease that is also a type 1 transmembrane protein, is the rate‐limiting and first proteolytic enzyme involved in the generation of Aβ‐peptide 5. Cleavage by BACE produces a large soluble ectodomain form of APP (β‐sAPP) and a membrane‐bound 99‐amino acid C‐terminal fragment (C99). Subsequently, the γ‐secretase protein complex cleaves the C‐terminus of C99 to produce a 40‐ to 42‐amino acid Aβ‐peptide 6. The cellular localization of this processing, particularly the interaction between APP and BACE, has been a focus of research in recent years. Following their synthesis in the endoplasmic reticulum, APP and BACE are transported via the Golgi apparatus. From the plasma membrane, these proteins pass through endocytotic pathways. BACE cycles between the plasma membrane, the endosomes and the trans‐Golgi network (TGN) until it is degraded in lysosomes. In contrast, APP is processed by various secretases (for review, see 7, 8, 9). However, the subcellular location of Aβ‐peptide generation has remained elusive. It was recently postulated that the generation of Aβ‐peptide occurs predominantly during anterograde transport, specifically in the Golgi apparatus or the TGN 10, 11. In neurons, APP is trafficked via synaptic vesicles to the synaptic surface 10, although its subsequent fate at the synapse remains a subject of current research. However, there is convincing evidence that APP must go through the endocytotic pathway to become fully processed 11 and that APP cleavage by BACE takes place in early endosomes 12, 13. Recent studies in neurons have shown recycling endosomes as sites where APP is processed by BACE 14, 15, 16. These pieces of evidence are bolstered by the finding that the acidic milieu of intracellular organelles supports the enzymatic activity of BACE 17. Nevertheless, following the inhibition of endocytosis, some BACE activity and Aβ‐peptide generation remain 18. The non‐amyloidogenic cleavage of APP by α‐secretase is considered to be predominantly localized to the cell surface 19. Although investigations into the trafficking pathways of APP and BACE have revealed that these two proteins are trafficked via separate endocytotic pathways, the relationship between the exocytosis of APP and BACE has not previously been addressed. However, because exocytosis is a key mechanism by which cells regulate their metabolism, it is important to clarify how these proteins reach the cell surface.

In this study, we investigated the distinct exocytosis characteristics of APP and BACE via simultaneous dual‐color total internal reflection fluorescence (TIRF) microscopy using HeLa cells transfected with pH‐dependent fluorescent proteins. Furthermore, we pharmacologically modulated the frequency of these fusion events, which represents a potential strategy to inhibit Aβ‐peptide generation.

## Results

### pHAPP and pHBACE are processed, transported and localized similarly to their endogenous counterparts APP and BACE

To visualize exocytosis, plasmids expressing APP and BACE fused to superecliptic pHluorin via their extracellular domains, thus preserving their signal peptides 10, were generated (pHAPP and pHBACE, respectively). pHluorin is a pH‐dependent green fluorescent protein (GFP) variant that is fluorescent in an environment with a neutral pH of 7.4 but is quenched in an acidic milieu 20. In this study, HeLa cells were transfected with pHAPP or pHBACE 24 h prior to experimentation.

**Figure 1:**
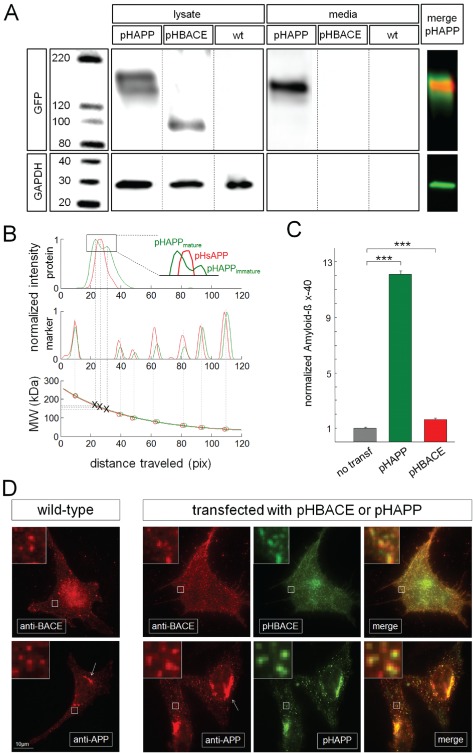
**pHAPP and pHBACE are processed, transported and localized similarly to their endogenous counterparts APP and BACE.** A) Western blots of the lysates and media of cells cultured for 48 h and transfected with pHAPP or pHBACE or untransfected (wt). An immunoblot of a pHAPP‐transfected cell lysate displayed an ∼170 kDa band, corresponding to mature pHAPP, and an ∼140 kDa band, corresponding to immature pHAPP. For the pHBACE‐transfected cell lysates, an ∼80 kDa band corresponding to pHBACE was detected. The loading control (GAPDH) was detected in all cells. An ∼155 kDa band, corresponding to pHsAPP, was detected in the pHAPP cell media. The images of the blots of pHAPP in the lysate (green) and the media (red) are merged. B) Western blot intensity profiles of the bands and molecular weight markers in the pHAPP‐transfected cell lysate (green) and media (red) according to the distance traveled. The bottom graph shows the plotted and interpolated molecular weight marker bands according to their associated molecular weights. Based on this interpolated curve, the exact molecular weights of mature pHAPP (168.6 kDa), immature pHAPP (144.1 kDa) and pHsAPP (158.9 kDa) were determined. C) The normalized (1 = 10.81 pg/mL) concentration of Aβ_x–40_ in the cultured HeLa cell media at 48 h. The Aβ_x–40_ concentrations in untransfected controls and pHAPP‐ or pHBACE‐transfected cells, respectively, are depicted. These measurements were performed via particle sorting‐based multiplexing. Overexpression of either protein caused a significant increase in the Aβ_x–40_ concentration compared to non‐transfection [Aβ_x–40untransfected_ = 10.81 pg/mL (±0.50 pg/mL), Aβ_x–40pHAPP_ = 134.73 pg/mL (±2.52 pg/mL), Aβ_x–40pHBACE_ = 17.62 pg/mL (±0.84 pg/mL); p_TTEST(pHAPP)_ = 9.0 × 10^−11^, p_TTEST(pHBACE)_ = 1.9 × 10^−5^, N = 8]. D) Representative immunocytochemistry images of untransfected and pHAPP‐ or pHBACE‐transfected HeLa cells labeled using an anti‐BACE or anti‐APP antibody, respectively. The cells were imaged via TIRF microscopy. APP and BACE were predominantly detected as clusters in both the untransfected and transfected cells, as shown in greater detail in the insets. The number of clusters per 3 µm‐sided square was not significantly different between the transfected and untransfected cells [number_wtAPP_ = 5.85 (±0.45), number_pHAPP_ = 5.25 (±0.22), p_TTEST(APP)_ = 0.19; number_wtBACE_ = 5.96 (±0.68), number_pHBACE_ = 6.24 (±0.65), p_TTEST(BACE)_ = 0.40; N = 5]. Examples of the colocalization of immunolabeling and transfected expression protein fluorescence are shown in the insets [Mander's overlap_pHBACE_ = 0.97 (±0.01), Mander's overlap_pHAPP_ = 0.97 (±0.01); N > 3]. The arrows indicate the annular accumulation of APP.

Prior to using pHAPP and pHBACE to examine the APP and BACE pathways, we confirmed that overexpressed pHAPP and pHBACE were processed and transported in manners similar to those of endogenous APP and BACE, respectively. The expression of both proteins was verified via western blot using a polyclonal GFP antibody (Figure 1A). pHAPP and pHBACE were only detectable via their pHluorin tags in the transfected cells. As expected, glyceraldehyde 3‐phosphate dehydrogenase (GAPDH) was present in all of the obtained lysates. The sizes of the posttranslationally modified mature APP (∼130–140 kDa), immature APP (∼110 kDa) and pHluorin (27 kDa) have been reported previously 20, 21, 22. Immunoblot analysis of the lysates of the pHAPP‐transfected cells revealed the expected bands for mature and immature pHAPP, suggesting the appropriate expression and maturation of pHAPP. To quantify the molecular weights (MWs) of the proteins detected in the pHAPP‐transfected cells, the intensities of the MW marker bands and the protein bands detected in the lysates and the media were plotted as intensity profile spectra according to the distance traveled (Figure 1B). Then, the distances traveled by the MW marker bands according to their MWs were plotted and interpolated via exponential fitting, which provided the exact MWs of mature and immature pHAPP and pHsAPP (Figure 1B, bottom graph).

When the proteins in the media of the cells were subjected to western blot, only an ∼155 kDa band corresponding to pHsAPP [size_sAPP_: ∼120 kDa 21] was detectable, which was expected because of the processing of APP by α‐secretase and the subsequent secretion of sAPP into the media. Based on exponential fit‐based size determination and image overlay analysis, the sAPP band in the media sample was determined to reside between the bands corresponding to the mature and immature forms of APP in the lysate sample (Figure 1B). With respect to BACE, we detected an ∼80 kDa band that corresponded to the sum of sizes of immature BACE [∼51 kDa 23] and pHluorin, indicating the appropriate expression of pHBACE. Both pHBACE and GAPDH, which are not secreted by living cells, were absent from the media, indicating that the cells were healthy.

These results demonstrating proper intracellular sorting and processing of pHAPP and pHBACE were expected, as the signal peptides of the utilized expression proteins were preserved. Additionally, quantitative analysis of the lysates revealed equal expression levels of total pHAPP and total pHBACE in the respective transfected cells [normalized intensity_pHAPP_ = 4.59× GAPDH (±0.50×), normalized intensity_pHBACE_ = 3.46× GAPDH (±0.27×), p_TTEST_ = 0.18; N = 3].

Appropriate processing of pHAPP was also confirmed based on measurements of the Aβ‐peptide forms in the media via particle sorting‐based multiplexing 24. The cells were cultured and either left untransfected or transfected with pHAPP or pHBACE. Measurements of Aβ‐peptide expression revealed that Aβ_x–40_ served as an appropriate marker to control for Aβ‐peptide generation in HeLa cells because it was properly detected in both the transfected and untransfected cells (data not shown). The presence of Aβ_x–40_ in the media of the transfected cells indicated that pHAPP was processed similarly to wild‐type APP and that pHBACE was cleaved at the same site as wild‐type BACE (Figure 1C). The levels of Aβ_x–40_ in the media of the transfected cells were increased more than 12‐fold in pHAPP‐transfected cells and more than 1.5‐fold in pHBACE‐transfected cells compared with the untransfected control cells, suggesting that the overexpression of both APP and BACE led to increased Aβ_x–40_ generation.

To compare the cell surface localization patterns of endogenous APP and BACE with those of pHAPP and pHBACE, respectively, transfected and untransfected HeLa cells were incubated with polyclonal antibodies against the C‐terminus of APP and BACE. Following incubation with Alexa568‐labeled (Invitrogen [Taufkirchen, Germany]) secondary antibodies, the cells were imaged via dual‐color TIRF microscopy to visualize both the secondary antibodies and the pHluorin‐tagged proteins. Both untransfected and transfected cells displayed agglomerations of APP and BACE at the cell surface and beneath the cell membrane to a depth of ∼100 nm (Figure 1D). These protein clusters are typically detected in fluorescence images of intracellular vesicles and organelles for proteins that accumulate at the cell surface. Such accumulations occur in clathrin‐coated pits during endocytosis or kiss‐and‐run exocytosis 25, 26, 27. To compare the untransfected and transfected cells, square regions of interest (ROIs) with a side length of 3 µm were randomly distributed on the cells, and the number of clusters was counted in each ROI. Neither pHAPP nor pHBACE overexpression led to a difference in the number of clusters compared with the endogenous expression of APP and BACE (Figure 1D, insets). Furthermore, both the pHAPP‐transfected and ‐untransfected cells displayed annular enrichment of APP expression (Figure 1D, arrows) based on APP immunolabeling. This enrichment of APP expression was closely associated with the Golgi apparatus, as the enrichment was localized annularly around the nucleus, which has been reported in previous studies 14. The colocalization of the anti‐BACE antibody and pHBACE and the anti‐APP antibody and pHAPP (Figure 1D, insets) confirmed that the overexpression of pHAPP and pHBACE did not induce any major structural changes or improper localization.

Together, these results confirm that pHAPP and pHBACE are trafficked similarly to endogenous APP and BACE, respectively, and that pHAPP and pHBACE are appropriate constructs for investigating the APP and BACE trafficking pathways.

### Exocytosis of pHAPP and pHBACE is clearly visualized via TIRF microscopy

Although many studies have investigated the intracellular trafficking of APP and BACE using various fluorescence assays, the characteristics of the exocytosis of these proteins have not been compared. Therefore, we sought to use established tools to examine the exocytosis of APP and BACE.

TIRF, or evanescent wave microscopy, is a technique that is extensively applied to investigations of the exocytosis of fluorescently tagged proteins. By illuminating the fluorescent probe at an angle above the critical angle, selective imaging of the plasma membrane and intracellular structures within a penetration depth of ∼100 nm from the glass coverslip is possible 28. Thus, events at the cell surface are not superimposed with intracellular signals, which occur when performing classical epifluorescence microscopy. The use of GFP as a fluorescence marker for the detection of exocytosis is possible in principle and has been successfully implemented in TIRF microscopy analyses 25, 29. However, if a protein does not display the characteristic properties of full‐collapse exocytosis, such as lateral diffusion in the membrane following exocytosis, but rather remains at the fusion site, the exocytosed protein and intracellular vesicles are indistinguishable because they emit nearly identical fluorescence intensities. The mechanism and performance of exocytosis cannot be assumed a priori. Therefore, a new fluorescent probe was used. The imaging of exocytosis was improved using pH‐dependent constructs that display increased fluorescence intensity when they contact the alkaline extracellular medium 20. This method has previously been performed to measure exocytosis and endocytosis by fusing the pH‐dependent GFP ‘pHluorin’ to the SNARE protein synaptobrevin 2 (this construct is known as synapto‐pHluorin or SpH) or to the transferrin receptor (TfR) 25, 30.

**Figure 2:**
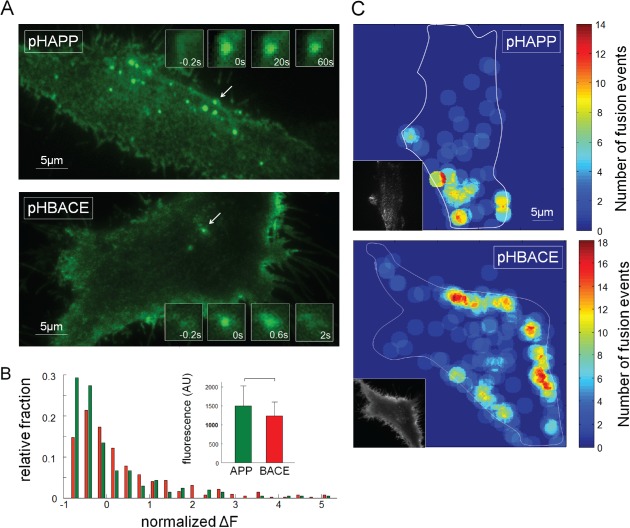
**Exocytosis of pHAPP and pHBACE is clearly visualized via TIRF microscopy.** A) HeLa cells transfected with pHAPP or pHBACE were imaged via TIRF microscopy over a 1‐min period at a frame rate of 5 Hz. Representative images of pHAPP and pHBACE from the image stacks are shown. The exocytosis of vesicles containing pHAPP or pHBACE appeared in the form of spontaneous, restricted increases in fluorescence. The arrows indicate characteristic fusion events, which are shown at the indicated time points (insets). B) The distribution of the amplitudes of the fluorescence increases that were observed for the pHAPP and pHBACE fusion events and the mean amplitudes (inset) (p_rank sum_ = 0.29, N = 10). The increases in fluorescence were normalized to the corresponding average value for comparison. C) Local distribution of fusion events within a 1‐min period (r_circles_ = 2.0 µm). The exocytosis sites were not randomly distributed, but multiple vesicles fused in succession at the same site or in close proximity. To identify the location of the fusion events with respect to the cell structure, the contour of each cell was delineated in each image. TIRF images of each cell are shown in the insets.

Therefore, we transfected HeLa cells with pHAPP or pHBACE (see also Movies S1 and S2, Supporting Information) and imaged the cells on the following day via TIRF microscopy. Figure 2A shows characteristic TIRF images of HeLa cells transfected with pHAPP or pHBACE. As expected, the release of pHAPP and pHBACE to the cell surface appeared as separate, specific events that were characterized by spontaneous, sharp increases in fluorescence in the regions involved. These results are consistent with the literature characterizing the use of TIRF microscopy to detect the exocytosis of fluorescently tagged proteins (for review, see 28).

Investigation of the increases in the fluorescence intensity in exocytotic regions revealed no significant differences between pHAPP‐ or pHBACE‐transfected cells (Figure 2B), indicating that equivalent levels of the fluorophores and, thus, the proteins per exocytosed vesicle were released. The distributions of the fluorescence intensity for both pHAPP and pHBACE were positively skewed (Figure 2B). This type of skewing has also been reported for synapses of SpH‐transfected neurons and is typical for vesicular proteins 31. Examination of the movies revealed that exocytosis occurred at preferred sites rather than at randomly distributed sites. Furthermore, multiple successive vesicles fused at the same site or in its close environment, which has previously been reported for other endocytotic events 25. To quantify these findings, 1‐min recordings at a frame rate of 5 Hz were analyzed, and the fusion events were automatically detected and counted using matlab software. Representative distributions of the number of fusion events per area are depicted in Figure 2C. Interestingly, the maximum number of fusion events per field per cell ranged from 6 to 24 for BACE [mean_BACE_ = 14.43 (±4.17)], whereas that for APP ranged from 5 to 55 [mean_APP_ = 26.89 (±9.31)] (*N* > 8). The detection of preferential exocytosis sites of APP and BACE based on this analysis may have been caused by technical artifacts due to the loss of adhesion of regions of the cells to the coverslip. Nevertheless, we detected this heterogeneous distribution in all of the analyzed cells. Furthermore, we would expect that a cell that lost contact with the coverslip would display no detectable fusion rather than a reduced fusion rate due to the low penetration depth of the evanescent field at these sites. A more likely explanation may be the proximity of these regions to the Golgi apparatus and the resulting short distances to the cell surface, resulting in a high fusion probability at these locations. The overwhelming presence of fusion events at the cell periphery might also have been caused by the excitation of both the upper and the lower plasma membranes by the evanescent field. However, in this circumstance, we would expect to observe this effect around the entire cell rather than at specific sites. Nevertheless, other cell types, such as neurons, immunological synapse‐forming cells and polarized epithelial cells, also contain specialized regions of exocytosis at the cell surface 32.

### The APP and BACE exocytosis events display distinct kinetic features

**Figure 3:**
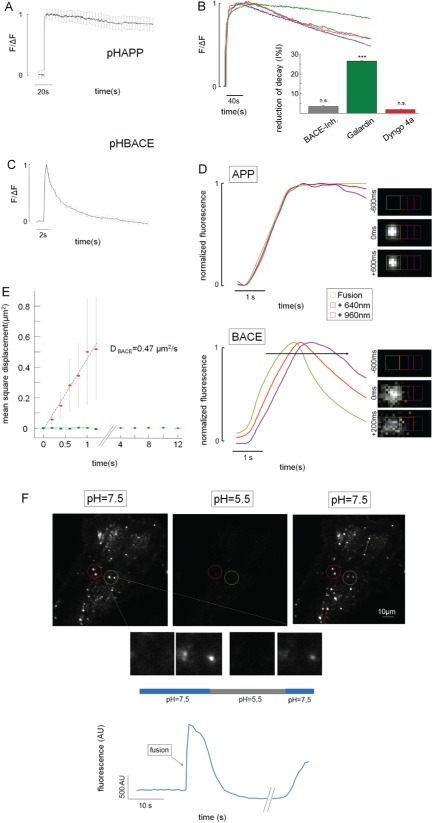
**The APP and BACE exocytosis events display distinct kinetics.** A) Normalized average fluorescence profiles of pHAPP exocytosis based on TIRF microscopy [τ_pHAPP_ = 125.3 seconds (±23.8 seconds), N > 5]. The curve is corrected for bleaching. B) Influence of various inhibitors on the fluorescence decay after pHAPP‐containing vesicle fusion events. The normalized average fluorescence profiles of cells treated with DMSO (blue), Galardin (green), Dyngo 4A (red) and the BACE inhibitor (gray) are shown after correction for bleaching. The bar graph displays the relative reductions in the decay rate based on the linear fit to the fluorescence profile after the fusion events for each inhibitor compared to the DMSO control. The BACE inhibitor reduced the linear decay rate by ∼3.48% (±0.15%); Galardin reduced the decay rate by ∼26.38% (±0.04%); and Dyngo 4A reduced the decay rate by ∼1.75% (±0.11%) (N > 3). Only the inhibitory effect of Galardin was significant (p_BACE‐Inh._ = 0.38, p_Galardin_ = 7.05 × 10^−5^, p_Dyngo 4A_ = 0.56). C) Normalized average fluorescence profiles of pHBACE exocytosis based on TIRF microscopy (N > 5). The fluorescence profile displayed different kinetics from pHAPP [τ_pHBACE_ = 2.28 seconds (±0.48 seconds); p_TTEST_ = 1.10 × 10^−8^; n > 8]. D) Normalized average fluorescence profiles of the ROIs centered at the fusion site and their corresponding neighboring regions in the pHAPP‐ and pHBACE‐transfected cells (n = 20). The fluorescence profiles were normalized to evaluate the delay in the increases in fluorescence. Relative to the amplitude of the fluorescence increase in the central ROI for pHAPP or pHBACE, those of the first neighboring ROIs (red plot) were ∼10% for pHAPP and 25% for pHBACE, and those of the second neighboring ROIs (purple plot) were ∼2.5% for pHAPP and 12.5% for pHBACE. E) The MSD for pHAPP (green dots) and pHBACE (red dots). The slope of the linear fit (gray line) corresponds to the diffusion coefficient (D_BACE_ = 0.47 µm^2^/second, n > 20). F) HeLa cells transfected with pHAPP were perfused with a pH 7.5 extracellular solution for 30 seconds, followed by perfusion with a pH 5.5 solution for 20 seconds and a final perfusion with a pH 7.5 solution for 10 seconds. The circles indicate fusion events that withstood the acidic pulse, whose appearances in the different phases of the experiment are depicted below. The arrows indicate fusion events during the acidic pulse. Bottom: the mean fluorescence profile of the fusion events, as indicated by the green circle, is shown.

Visual examination of the fusion events suggested different kinetics for the exocytosis of APP and BACE. Therefore, we characterized the kinetic properties of APP and BACE exocytosis based on their fluorescence profiles. To investigate the fluorescent exocytosis signals corresponding to pHAPP and pHBACE, the fusion events were manually selected, synchronized to the time of fusion and averaged. The averaged profiles of the pHAPP and pHBACE fusion events revealed sudden, rapid increases in fluorescence (Figure 3A,C). While the pHBACE fusion events were immediately followed by an exponential rapid decrease in fluorescence [*τ*_pHBACE_ = 2.28 seconds (±0.48 seconds)], the fluorescence corresponding to the pHAPP fusion events decreased very slowly in a nearly linear manner [*τ*_pHAPP_ = 125.3 seconds (±23.8 seconds)].

Consequently, we designed experiments to clarify the mechanisms underlying these different kinetic profiles. First, the slow, continuous decreases in the pHAPP fluorescence profiles may have been due to the direct processing of pHAPP by α‐secretase or BACE, which would deplete the fluorescence intensity if the proteins subsequently diffused away. Both α‐secretase and BACE are located on the cell surface 19, 33. Therefore, we incubated pHAPP‐transfected HeLa cells in a BACE inhibitor or dimethyl sulphoxide (DMSO) for 30 min before performing the TIRF microscopy measurements. To quantify the effect of the BACE inhibitor on the fluorescence decay profiles, we linearly fitted the fluorescence profiles after the respective fusion events and compared the calculated decay rates (Figure 3B). Interestingly, the BACE inhibitor caused a reduction in the mean decay rate of only 3.48% (±0.15%), which is consistent with the common perception that the processing of APP by BACE primarily occurs in intracellular compartments (for review, see 34 35). To inhibit α‐secretase, we incubated the transfected cells in the metalloproteinase inhibitor Galardin 36, 37 or DMSO for 30 min. Because of the reduced activity of α‐secretase, the mean fluorescence decay rate of the Galardin‐treated cells was reduced by 26.38% (±0.03%) compared with that of the DMSO‐treated control cells (Figure 3B). This clear reduction in the fluorescence decay provides optical evidence that the cleavage of APP by α‐secretase occurs on the cell surface as previously described 19.

Furthermore, endocytosis of APP at the same site might potentially explain the observed decrease in fluorescence. We incubated HeLa cells in Dyngo 4A (Abcam [Cambridge, UK]) for 30 min to inhibit dynamin and, therefore, endocytosis 38 as demonstrated by the detection of the reduced uptake of transferrin‐Alexa 594 (Figure S1). Treatment with Dyngo 4A caused a reduction in the pHAPP fluorescence decay rate of only 1.75% (±0.01%). Thus, pHAPP (full length) is likely not endocytosed at the same site as at which exocytosis occurs (Figure 3B).

Finally, slow decreases in the fluorescence profiles of the pHAPP‐containing vesicles are also caused by photobleaching. We probed this phenomenon by switching the excitation intensity from 3.15 to 12.6% of the maximum laser intensity, which resulted in a change in the mean calculated τ value from 101.7 seconds (±25.4 seconds) to 48.6 seconds (±8.6 seconds) (N = 3). In the case of pHBACE, converting the excitation intensity from 3.15 to 12.6% or 100% of maximum laser intensity did not result in a significant change in the τ value [τ_pHBACE_100_ = 5.42 seconds (±0.52 seconds), τ_pHBACE_12.6_ = 4.03 seconds (±0.79 seconds), τ_pHBACE_3.15_ = 3.60 seconds (±0.52 seconds); Kruskal–Wallis test: χ ^2^_2,56_ = 4.5, p = 0.11; n = 19]. Thus, because bleaching is a relevant factor, all comparative measurements were performed under the same conditions (e.g. exposure time, filter and laser intensity) and were corrected for bleaching 10.

Exponential signal decreases similar to those detected for pHBACE following fusion events have been linked to lateral diffusion following exocytosis 28, 39. This explanation is in accordance with our visual examination of the video recordings obtained in this study, in which we observed diffuse clouds after exocytosis similar to those that have been described in previous studies 28, 39, 40. Thus, we examined the lateral diffusion of the exocytosed proteins in detail.

To this end, we defined square ROIs with a side length of 640 nm around the fusion events of vesicles containing either pHAPP or pHBACE. Additionally, the intensities in regions separated by 320 nm were analyzed. During fusion, the expected time course of fluorescence was detected in the ROIs located directly at the fusion site. Additionally, a minor change in the fluorescence intensity was detected in the respective neighboring regions, which was caused by slight irradiation of the light from the central ROI. Figure 3D shows the normalized fluorescence profiles of the central ROI (green plot) and the respective neighboring ROIs (red and purple plots). Notably, the amplitudes of the fluorescence profiles were normalized to assess the time points in which the fluorescence intensity increases were initiated. If lateral diffusion occurs after the fusion events, we would expect to detect both of the following characteristics: the longest delay in the increase in fluorescence and the point at which that increase reached its maximum should occur in the most distant ROI. For the pHAPP fusion events, there were no detectable delays in the increase in fluorescence in the adjacent ROIs, suggesting that no lateral diffusion occurred after exocytosis (Figure 3D). In contrast, following pHBACE fusion events, the fluorescence intensity was found to spread with a clearly increasing time delay at larger distances from the initial fusion site; for example, a delay in the fluorescence increase of 200 milliseconds was detected 640 nm from the fusion site, implying lateral diffusion (Figure 3B). To quantify these findings, the fusion events were fitted with two‐dimensional (2D) Gaussians centered on the maximal fluorescence increase, and the detected radius of the 2D Gaussian fit was used to calculate the mean square displacement (MSD) (Figure 3E). As expected, following the exocytosis of pHBACE, the MSD increased continuously (red dots), and linear fitting produced a diffusion coefficient of D_BACE_ = 0.47 µm^2^/second. In contrast, there was no detectable MSD following the exocytosis of pHAPP (green dots).

The lack of lateral diffusion of pHAPP raised the question of whether APP‐carrying organelles fuse with the membrane at all or whether the detected fluorescence increases represented sudden changes in organelle pH that were independent of surface contact. The latter phenomenon would exclude the possibility of any interaction between APP and BACE at the plasma membrane after fusion. To determine whether the detected fluorescence increases represented organelle fusion with the plasma membrane, we performed an experiment in which the pH‐dependent cell surface fluorescence was quenched by applying an acidic solution at a defined time point. If the previously described fluorescence increases represent intracellular events, e.g. instant alkalization of organelles, these increases would be expected to withstand such a surface‐quenching procedure.

HeLa cells transfected with pHAPP were perfused with a modified extracellular solution equilibrated to pH 7.5, and the cells were imaged via TIRF microscopy. After 30 seconds, the extracellular solution was replaced with a pH 5.5 solution and was replaced again with the pH 7.5 solution after another 20 seconds. During perfusion with the acidic extracellular solution, the fluorescence of the exocytosed pHAPP was quenched. The fluorescence increased again following replacement with the neutral pH solution. The finding that the formerly strongly fluorescent organelles were quenched during the initial solution exchange procedure and regained fluorescence upon the conversion to the neutral pH solution demonstrated that pHAPP reached the cell surface and that the changes in pHAPP fluorescence were indicative of the exocytosis of pHAPP. A possible kiss‐and‐stay mechanism, characterized by a restricted open fusion pore that closes very slowly 41, cannot be excluded for these APP‐carrying organelles.

### Both APP‐ and BACE‐containing vesicles display characteristic docking during fusion

Exocytosis can be separated into three steps: transport of the organelles to the cell membrane, docking at the membrane and fusion with the cell membrane. Each of these steps can be detected via TIRF microscopy (for review, see 28). Therefore, we next examined whether APP‐ and BACE‐containing vesicles also underwent these steps. This assessment required the visualization of intracellular structures because steps one and two occur intracellularly.

**Figure 4:**
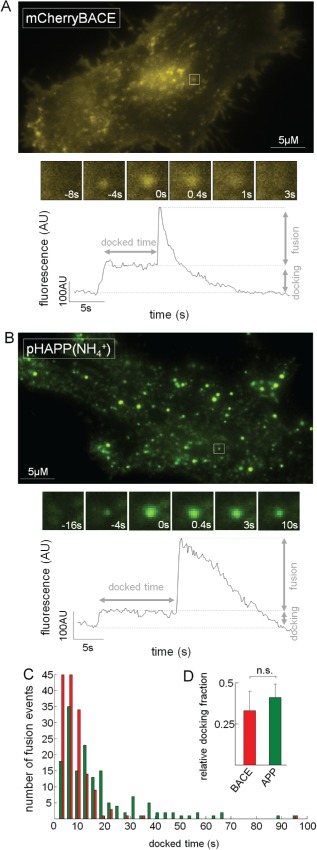
**Both APP‐ and BACE‐containing vesicles display characteristic docking during fusion.** A) HeLa cells transfected with pH‐independent mChBACE were examined via TIRF microscopy according to our standard protocol. A representative image of one of these video recordings is shown. The rectangle indicates a typical fusion event, the appearances of which at different time points are depicted in the insets. Prior to fusion (time = 0 second), a strongly fluorescent spot was already detectable at the fusion site. The fluorescence profile of this region displayed not only the typical kinetics of BACE fusions but also an increase in fluorescence (docking) with a subsequent plateau prior to fusion. Notably, mChBACE displayed reduced contrast due to the lateral diffusion of BACE. B) HeLa cells transfected with pHAPP were imaged via TIRF microscopy in an extracellular solution containing NH_4_^+^. Representative images obtained from a video recording of the appearance of a characteristic fusion event (rectangle) at different time points are shown. The fluorescence profile reveals the docking and other typical characteristics of APP fusion events. C) The histogram shows the distribution of the docking times of pHAPP‐ and mChBACE‐containing vesicles prior to fusion (N > 5). D) Bar graph depicting the ratio of fusion events that had previously undergone docking to all fusion events (p_TTEST_ = 0.39; N > 5).

Because BACE fusion events can be unambiguously discerned due to the protein's lateral diffusion following exocytosis, we assumed that these fusion events could also be detected using a pH‐independent fluorophore, providing us with the advantage of also observing intracellular organelles. Therefore, we transfected HeLa cells with BACE fused to mCherry instead of pHluorin and imaged the cells via TIRF microscopy according to our protocol. Figure 4A shows a representative TIRF image of a HeLa cell transfected with mCherry‐BACE (mChBACE). As observed in the pHBACE recordings, the edges and filopodia of the cells were clearly visible. Additionally, intensely fluorescent moving and resting spots were detected that corresponded to intracellular mChBACE‐carrying organelles and extracellular clusters of mChBACE, respectively. Furthermore, fusion events were detectable based on lateral diffusion as described previously. The rectangle in Figure 4A indicates one of these fusion events, the appearance of which is depicted at different time points in the insets. Interestingly, the fusing organelle had entered the evanescent field several seconds before fusion and then remained docked at the fusion site. The fluorescence profile of this fusion event confirmed the visual observations. Prior to the initiation of the characteristic kinetic profile of the BACE fusion event, there was a fluorescence increase followed by a plateau, which was caused by the docking of the organelle. The further increase in fluorescence during fusion was caused by the reduced distance between the fluorophores and the glass coverslip; this reduced distance facilitated more effective excitation of the fluorophores due to the exponential nature of the evanescent field in TIRF microscopy 42.

To analyze the intracellular movements and the docking of pHAPP‐containing organelles, HeLa cells transfected with pHAPP were imaged in an extracellular solution containing NH_4_^+^, which results in the alkalization of intracellular vesicles and organelles and the fluorescence of intracellular pHAPP 43. A representative TIRF image showing a cell containing several distinct spots is shown in Figure 4B. Again, the fusion events appeared as bright, localized spots and were detectable because of the sudden increase and subsequent decrease in fluorescence. The insets show a representative fusion event, indicated by a rectangle in the TIRF image. It should be noted that, consistent with the above results, no diffusion was visible. The fluorescence profile of this ROI exhibited the typical kinetics of an APP fusion event. Again, the organelle‐docking step was observed prior to the ultimate fusion. These docking steps for both pHAPP and mChBACE were indicative of exocytotic mechanisms typical for the respective proteins. We analyzed the durations of the docked statuses of various organelles (Figure 4C). The docking times ranged from 1.60 to 34.40 seconds for BACE‐containing organelles [mean docked time_BACE_ = 8.34 seconds (standard deviation: ±8.80 seconds)] and from 0.92 to 45.77 seconds for APP‐containing organelles [mean docked time_APP_ = 8.59 seconds (standard deviation: ±7.77 seconds)]. The docked times of both APP‐ and BACE‐containing organelles displayed Poisson distributions, which may also suggest the existence of events that are too fast to detect 44.

However, only 32.72% (±11.52%) and 41.19% (±7.59%) of all fusion events detected in mChBACE‐ and pHAPP‐transfected cells, respectively, displayed detectable docking using our method (Figure 4D). The other fusion events displayed kinetic profiles that were similar to those of the pHBACE and pHAPP fusion events when only the cell surface was examined. These numbers contrast with those reported in another TIRF study that investigated the exocytosis of insulin; this study found that the majority of insulin secretion events occur from previously docked granules 45, and this finding may be due to differences between constitutive and regulated exocytosis.

### pHAPP and pHBACE are exocytosed in spatially and temporally distinct manners

**Figure 5:**
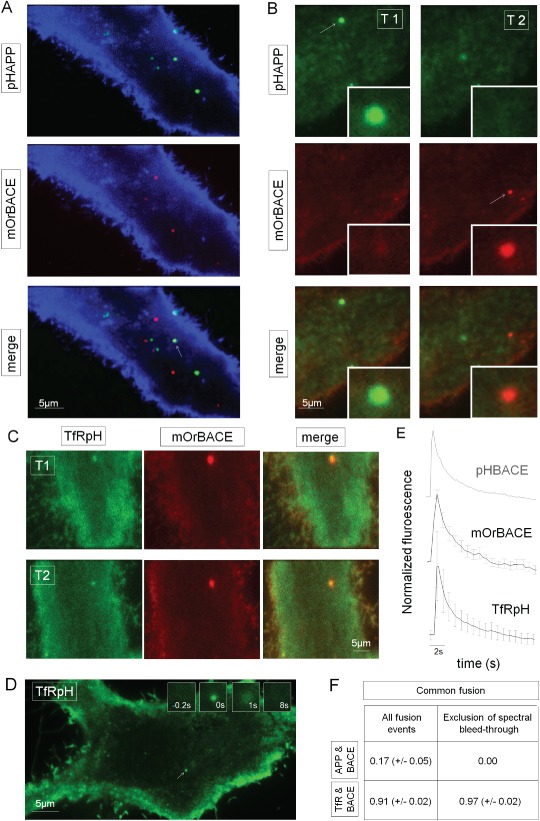
**pHAPP and pHBACE are exocytosed in spatially and temporally distinct manners.** A) Maximum intensity projection of the difference images of a HeLa cell co‐transfected with pHAPP (green) and mOrBACE (red). The cells were simultaneously imaged in two fluorescence channels for a period of 1 min via TIRF microscopy. The minimum projection of mOrBACE (blue colored) was used to visualize the cell. B) The arrows show pHAPP and mOrBACE fusion events at different time points (T1 and T2, respectively). C) Fusion events in a representative HeLa cell co‐transfected with TfRpH and pHBACE. The exocytosis of TfRpH colocalized with that of mOrBACE. D) Representative TIRF image of a HeLa cell transfected with TfRpH. The arrow indicates the fusion event whose appearance at different time points is shown in the insets. E) Normalized average fluorescence profiles of TfRpH (n > 40) and mOrangeBACE (n > 15) fusion events. For comparison, the normalized average fluorescence profile of pHBACE from Figure 2A is shown in gray. pHBACE, mOrBACE and TfRpH displayed the same exocytosis kinetics [τ_pHBACE_ = 2.28 seconds (±0.48), τ_mOrBACE_ = 2.08 seconds (±0.74), τ_TfRpH_ = 2.77 seconds (±0.29); Kruskal–Wallis test: χ ^2^_2,27_ = 1.37, p = 0.50; n = 10]. F) Table showing the spatially and temporally overlapping fusion events for pHAPP and pHBACE and for TfRpH and pHBACE (N > 10). Spectral bleed‐through was excluded by analyzing only the fusion events that were initially detected using the mOr channel.

The distinct exocytosis kinetics of pHAPP and pHBACE suggested that these two proteins are transported to the surface separately. To exclude the possibility of transport and exocytosis in a common vesicle, we performed simultaneous dual‐color imaging of APP and BACE. For this purpose, we exchanged the pHluorin tag of BACE with mOrange to generate mOrBACE. Similar to pHluorin, mOrange is pH‐dependent, but mOrange displays distinct spectral properties 46, which enabled us to simultaneously measure mOrange and pHluorin. mOrBACE displayed the same kinetic profile as pHBACE (Figure S2 and Figure 5E). The cells were imaged using a TIRF microscope equipped with a beam splitter that separated the emission wavelengths (Movie S2). Figure 5A shows the separate maximum projections of the difference images of cells co‐transfected to express pHAPP and mOrBACE. The minimum projection of mOrBACE was used to visualize cell contours and backgrounds. Only one pHAPP and mOrBACE colocalization event (Figure 5A, arrow) was detected in the merged image. However, analysis without the maximum projection over time revealed that this colocalization was temporally separated by more than 10 seconds (not shown). Figure 5B shows representative images from the image stack of another co‐transfected cell at two time points. Representative pHAPP and mOrBACE fusion events are depicted as insets, and these images confirm that there were no detectable fusion events in the other channels.

To compare these results with those for another transmembrane protein, we co‐transfected HeLa cells with mOrBACE and a TfR–pHluorin construct (TfRpH). Transferrin is a widely used model for analyses of vesicle recycling (for review, see 47 48). Additionally, TfR is assumed to colocalize with BACE in recycling vesicles in neurons 14. Interestingly, the patterns of exocytosis of TfRpH and mOrBACE were clearly spatially and temporally colocalized, which supports the hypothesis that these two proteins underwent cellular transport to the surface in common vesicles (Figure 5C). Next, we were interested in determining whether the kinetics of the exocytosis of TfRpH were the same as those of pHBACE. To exclude the possible influence of co‐transfection, HeLa cells were transfected with TfRpH alone and imaged via TIRF microscopy for further analysis. Visual inspection revealed similar observations to those from the pHBACE video recordings (Figure 5D). Detailed analysis of a characteristic fusion event (Figure 5D, arrow), whose appearance at different time points is shown in the insets, revealed that the fusion events also appeared as distinct, sudden increases in fluorescence that were followed by lateral diffusion; these findings are in accordance with those from the pHBACE analysis described above. The normalized averaged fluorescence profiles of the TfRpH fusion protein revealed identical kinetics to the pHBACE and mOrBACE fusion proteins (Figure 5E). Figure 5F shows the proportion of fusion events common to pHAPP and mOrBACE and to TfRpH and mOrBACE. The fusion events were manually selected in each fluorescence channel, and the other corresponding channel was then evaluated for possible simultaneous changes in fluorescence. Fusion events were recognized as increases in fluorescence intensity that exceeded two standard deviations above the background noise that displayed typical kinetics. Spectral bleed‐through, particularly from the green channel to the red channel, accounted for 11% of the total emissions and was therefore an important limiting factor. This bleed‐through could be excluded by analyzing only the fusion events that were primarily detected in the red mOrBACE channel because of the lack of bleed‐through from the red channel to the green channel. This analysis also enabled the exclusion of single events of TfRpH exocytosis, which resulted in a higher relative proportion of fusion events common to TfRpH and mOrBACE compared to the analysis in which bleed‐through was not excluded. These data clearly demonstrate that APP and BACE are exocytosed separately, in contrast to BACE and TfR.

### The numbers of pHAPP and pHBACE fusion events are distinct and alterable

Reaching a common compartment, e.g. the cell membrane, is a crucial requirement for BACE to process APP. Because APP and BACE are secreted separately, we sought to determine whether the secretion of both proteins is pharmacologically alterable. Therefore, we examined the frequency of these fusion events and investigated their pharmacological regulation.

**Figure 6:**
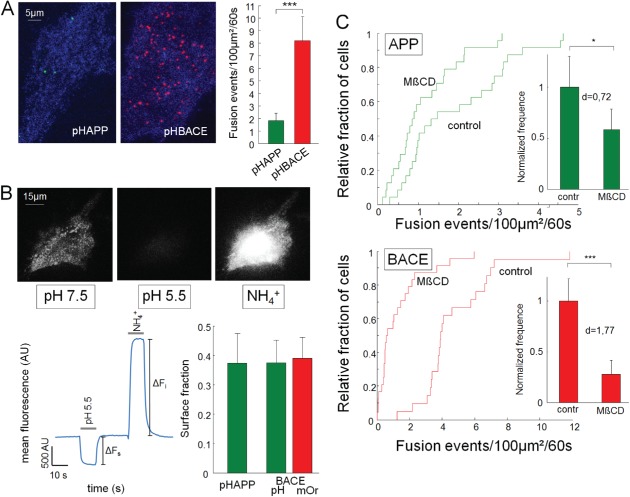
**The numbers of pHAPP and pHBACE fusion events are distinct and pharmacologically alterable.** A) Maximum intensity projection of the difference images of HeLa cells transfected with pHAPP or pHBACE. The cells were imaged for a 1‐min period at a frame rate of 5 Hz via TIRF microscopy. Minimum intensity projections (blue) were used to visualize the cells. Bar graphs depicting the mean number of fusion events per 100 µm^2^ within a 1‐min period (p_rank sum_ = 1.9 × 10^−7^, N > 20). B) HeLa cells transfected with pHAPP or pHBACE were perfused with a pH 7.5 extracellular solution. The perfusate was then converted to a pH 5.5 solution and finally converted to a solution containing NH_4_^+^ for 10 seconds during epifluorescence imaging. The fluorescence profile shows the mean fluorescence of a representative cell. The acidic and ammonium pulses and the amplitudes of ΔF_s_ and ΔF_i_ are included. The bar graphs show the resulting surface fractions of pHAPP, pHBACE and mOrBACE (p_TTEST(pHAPP,pHBACE)_ = 0.50; p_TTEST(pHBACE,mOrBACE)_ = 0.40; n > 15). C) The cumulative density functions of the number of fusion events in the untreated (control) and MβCD‐treated cells (N > 20). The bar graphs show the normalized mean number of fusion events per 100 µm^2^ of untreated (control) and MβCD‐treated cells within a 1‐min period (pHAPP: p_rank sum test_ = 0.02, d_Cohens_ = 0.72; pHBACE: p_rank sum test_ = 1.7 × 10^−6^, d_Cohens_ = 1.77; N > 20). The results are provided for pHAPP‐ and pHBACE‐transfected HeLa cells.

In addition to distinct kinetics, a difference in the frequency of fusion events was detectable based on visual examination. Figure 6A shows the maximum intensity projections of difference images of HeLa cells transfected with pHAPP or pHBACE. pHBACE clearly displayed a greater number of exocytosis events than pHAPP. To quantify the fusion events, the ROIs for specific fusion events were automatically defined from image stacks 49, and the resulting fluorescence profiles were then manually inspected to count the fusion events. The exocytosis of pHBACE‐containing vesicles occurred more than four times as often as the exocytosis of pHAPP‐containing vesicles (Figure 6A).

Because of this result and because vesicles contain the equivalent levels of the pHAPP and pHBACE proteins (Figure 2B), we examined whether the proportion of these proteins at the cell surface relative to the intracellular contents was equal for pHAPP and pHBACE. For this purpose, we used a protocol that was introduced by Sankaranarayanan et al. to calculate the ratio of surface protein to total protein 30. HeLa cells transfected with pHAPP, pHBACE or mOrBACE were perfused with different extracellular solutions of different pH and were imaged via epifluorescence microscopy. ROIs containing the entire cell were quantified, and the corresponding mean fluorescence profiles were analyzed. While perfusing the cells with a standard pH 7.5 solution, the pH‐dependent fluorophores tagged to surface proteins were fluorescent, whereas the fluorophores in acidic organelles were quenched. Exchanging the extracellular solution with a pH 5.5 solution quenched the surface protein fluorescence. Finally, perfusion with an extracellular solution containing NH_4_^+^ caused alkalization of all formerly acidic intracellular organelles, thus increasing the fluorescence intensity. The surface fraction was calculated as the ratio of Δ*F*_s_ to the sum of Δ*F*_s_ and Δ*F*_i_ (see Figure 6B). There were no significant differences between the surface fractions of pHAPP, pHBACE and mOrBACE. Based on our findings that the frequency of BACE fusion events was much higher than that of APP, whereas the amount of each protein per vesicle and the surface fraction of each protein were the same, there must have been more vesicle recycling of BACE than APP.

We next sought to determine whether the frequency of either pHAPP or pHBACE fusion was alterable. For this purpose, we used methyl‐β‐cyclodextrin (MβCD), which causes cholesterol depletion in the plasma membrane, thereby decreasing the extracellular Aβ‐peptide levels 33, 50, 51, 52. To evaluate the effects of MβCD on the exocytosis of APP and BACE, HeLa cells were transfected with pHAPP or pHBACE and incubated in MβCD for 45 min. In our experiments, MβCD reduced the frequency of the exocytosis of both pHAPP and pHBACE compared with the untreated controls. However, the reduction of pHBACE exocytosis was significantly greater than that of pHAPP (*p*_rank sum_ = 3.1 × 10^−3^). The MβCD‐induced alteration of postsynaptic membrane viscosity via cholesterol depletion has recently been shown to cause increased diffusion 53. Therefore, we analyzed lateral diffusion during fusion events in pHAPP‐transfected cells treated with MβCD. Again, no diffusion of APP was detected, despite pre‐treatment with MβCD (data not shown). These results demonstrated that the frequencies of both APP and BACE fusion are pharmacologically alterable.

## Discussion

In this study, we demonstrated that TIRF imaging of pHAPP and pHBACE can be used to evaluate the exocytosis of APP and BACE. We found that APP and BACE exhibit distinct fusion kinetics and surface trafficking. Furthermore, BACE and TfR are transported and released together, and the fusion frequencies of APP and BACE are distinct and can be pharmacologically altered using MβCD.

Our results are primarily based on the use of TIRF microscopy, which selectively images the 100 nm above the coverslip and, thus, the plasma membrane 54. The resulting high signal‐to‐noise ratio compared to that of epifluorescence microscopy renders TIRF microscopy as an appropriate tool for measuring exocytosis 28, 55. In observations of live cells, translationally fused probes label proteins from their synthesis onward, whereas affinity‐based probes only label proteins that have been exposed to the surface. This distinction is relevant if a protein is processed, thus removing some of its epitopes, after its initial exocytosis to the cellular surface, which may be the case for APP. Therefore, for live‐cell imaging of proteins that are in part newly synthesized, the transfection of cells using specific fluorescently tagged proteins is an appropriate method that has resulted in a plethora of important findings 25, 56. The problem of distinguishing between intracellular vesicles within 100 nm of the coverslip and proteins localized to the surface was circumvented via the use of pHluorin. Adverse effects of overexpression on trafficking pathways are often examined using this method. To reduce these possible effects, fluorophores were inserted between the signal peptides and the mature peptides of APP and BACE. Our data show that pHAPP is processed in a manner similar to that of endogenous protein (Figure 1A,B) and that transfection with pHBACE also leads to an increase in Aβ, which is indicative of proper processing (Figure 1C). Additionally, both pHAPP and pHBACE could be detected using specific antibodies, and the general distribution parameters (surface clustering) of APP and BACE were not changed by transfection (Figure 1D). However, the results of these immunocytochemical assays are limited in terms of the identification of the organelles in which the proteins show clusters of fluorescence.

We found that pHAPP and pHBACE exhibit different fluorescence time courses following surface fusion of the organelles carrying them. pHAPP fluorescence decreased linearly, whereas pHBACE fluorescence decreased exponentially. The slow, continuous fluorescence decay and the absence of lateral diffusion of APP suggest that APP remains at the same site after exocytosis. This conclusion is consistent with the notion that APP is enriched in cholesterol‐ and sphingolipid‐rich membrane microdomains (for a review, see Simons et al. 57). Furthermore, plasmalemmal clusters formed by APP are involved in APP trafficking and regulation, which is also in agreement with the finding that the APP fluorescence signal is stationary 50, 58. The linear decrease in pHAPP fluorescence was extensively investigated in this study. These investigations revealed that the decrease in fluorescence was primarily caused by the cleavage of APP by α‐secretase rather than β‐secretase or endocytosis (Figure 3B). Our findings completely concur with other reports that α‐secretase cleavage has been considered to be localized predominantly to the cell surface 19. Moreover, cleavage of APP by β‐secretase is thought to be an intravesicular process and, thus, is not expected to significantly influence the cleavage of APP from the cell surface 17. Nevertheless, we cannot exclude the possibility that APP is cleaved by BACE at other surface sites. Interestingly, in our experiments, the inhibition of endocytosis did not reduce the fluorescence decay rate following exocytosis, providing optical evidence that dynamin‐dependent endocytosis of full‐length APP (bearing the pHluorin) does not occur at the same sites as APP exocytosis. In contrast, exocytosed tissue‐type plasminogen activator (tPA)‐GFP proteins have been reported to remain at a particular location, suggesting resealing and re‐acidification as possible mechanisms underlying their lack of lateral diffusion 59. However, we found that the APP that was secreted to the cellular surface was accessible to the extracellular solution (Figure 3F). Nevertheless, we cannot exclude the possibility of APP performing kiss‐and‐stay or other dynamin‐independent maneuvers at the site of its exocytosis. Recent studies performing live‐cell TIRF microscopy in neurons indicated kiss‐and‐run as a persistent exocytotic pathway that is characterized by a slow decrease in the fluorescence of TfRpH 60, 61. In our study, we could not observe this slow decrease in TfRpH fluorescence, which may be due to the usage of different cellular systems, in particular neurons versus Hela cells. The lateral diffusion of individual fluorescent molecules, which is not detectable in our technical system, may further contribute to the decay of APP fluorescence.

In contrast to the fusion kinetics of APP, those of BACE are reminiscent of other exocytosis profiles. In the BACE kinetic profile, a rapid increase in fluorescence intensity is followed by an exponential decay that is typically associated with diffusion, and the diffusion coefficient was consistent with previous studies 62, 63. Such fusion activity has also been shown for VGLUT‐EGFP‐containing vesicles in astrocytes 64, for fusion events in Synapto‐pHluorin‐expressing astrocytes 39, for pancreatic β‐cells expressing Neuropeptide Y‐mRFP 40 and for VAMP8‐GFP‐expressing HeLa cells 29. Interestingly, these kinetics are typical of Ca^2+^‐dependent exocytosis 28 and may serve as a new approach for obtaining further insight into BACE exocytosis.

Furthermore, we demonstrated that BACE and TfR are exocytosed together; this finding is in accordance with other studies that have shown the colocalization of BACE and TfR in recycling vesicles in neurons 14. TfR is often used to analyze vesicle recycling and its fusion characteristics; for example, the regulation of vesicle recycling by the Rab11 GTPase 65 has been investigated in detail. Therefore, our findings support the hypothesis that BACE is transported via recycling endosomes and, therefore, that Rab11 regulates Aβ‐peptide production 16, 66.

Our findings that APP and BACE are exocytosed by distinct vesicles extends the understanding of their trafficking pathways, which have been postulated to play key roles in Aβ‐peptide generation [for review, see Sannerud and Annaert 8]. The secretory pathway, particularly the TGN, is considered as the primary pathway of Aβ‐peptide generation 67, 68. Based on this assumption, we would expect that some exocytotic fusion events would release both BACE and soluble sAPP. However, our measurements only detected the exocytosis of APP or BACE from individual organelles. Our results support the possibility of processing at the cell surface because after APP and BACE have entered the cell membrane, BACE diffuses and can therefore reach APP. However, as described above, the processing of APP by BACE was not the predominant event at the cell surface.

We found that APP and BACE are exocytosed from separate organelles, and we demonstrated that the trafficking of these proteins represents a possible target for therapeutic intervention because we could alter the frequency of fusion via pharmacological manipulation. Following treatment with MβCD, we found that the frequency of APP fusion events and, to a greater extent, the frequency of BACE fusion events were effectively decreased. It is unlikely that MβCD‐induced alterations in gene expression affected vesicular exocytosis in this study because of the short 45‐min incubation period we employed. It is more likely that MβCD disturbed the membrane lipid rafts within which APP and BACE are clustered with SNARE proteins; this disruption may then lead to decreased exocytosis 33, 69. This effect of MβCD on exocytosis has previously been demonstrated in pancreatic islet cells 70.

In summary, we show that APP and BACE reach the cell surface via distinct organelles prior to being cleaved via the non‐amyloidogenic pathway. Our results provide a conceptual basis for the neuronal secretion of these proteins. We have previously shown that APP is secreted by synaptic vesicles 10. Because synapses are nearly diffraction‐limited objects, in the current study, we utilized the spatial segregation of fusion events in tumor cells to address this issue. How APP and BACE reach the synaptic surface remains unclear. However, dynamic findings at synaptic sites can be interpreted in the context of the present results. The results based on fluorescently labeled APP only provide information about the labeled protein, which in the present study was at the N‐terminal of the protein. Our results are consistent with those of our previous study that the N‐terminus of APP is exocytosed from vesicles. However, only when fluorescent probes that facilitate the monitoring of Aβ‐peptide isoforms themselves become available will we be able to discover the complete trafficking cycle.

Furthermore, we show that secretion to the cell surface can be pharmacologically modulated. The influence of vesicle secretion may be a promising new and highly relevant protein‐independent target for therapeutic interventions and may prove valuable for improving our understanding of the involvement of cellular trafficking in Alzheimer's disease.

## Methods

### Cell culture and transfection

HeLa cells were maintained at 37°C in 5% CO_2_ in DMEM (Biochrom) containing 5 g/L glucose and supplemented with 10% FBS (Invitrogen), 1% glutamine (Biochrom) and 1% NEAA (PromoCell). The cells were harvested using trypsin (Invitrogen) and seeded on 10‐cm dishes (Biochrom).

For imaging, the cells were separated and seeded directly on glass coverslips in 12‐well dishes (Biochrom) 48 h before the experiments were performed. One day prior to the experiments, the cells were transfected with the indicated plasmids using polyethylenimine.

### Plasmid constructs, antibodies and reagents

Plasmids expressing the fusion proteins pHAPP, pHBACE, mOrBACE or mChBACE under the control of a CMV promoter were constructed via standard molecular biology methods using Synapto‐pHluorin 20, mOrange (Clontech), pmCherry (Clontech), BACE‐CFP and pHAPP 10 as templates for polymerase chain reaction amplification. The fluorophores were inserted between the signal peptide and the mature peptide of APP and BACE to ensure appropriate transmembrane localization of the proteins, with the fluorophores pointing toward the luminal/extracellular side. The sequences of any of these plasmids are available upon request. TfRpH was a kind gift from D. Perrais 25.

The use of the pH‐dependent GFP variant pHluorin 20 or the RFP variant mOrange2 46 fused to the luminal side of the respective proteins enabled the detection of membrane fusion based on the increase in fluorescence in alkaline media 20.

For western blot and immunofluorescence analyses, the following antibodies were used. A polyclonal rabbit anti‐GFP antibody was purchased from Life Technologies, and a monoclonal mouse anti‐GAPDH antibody was purchased from Merck Millipore. A polyclonal rabbit anti‐APP C‐terminal antibody was obtained from Synaptic Systems (SySy), and a polyclonal rabbit anti‐BACE1 C‐terminal antibody was purchased from Sigma‐Aldrich. The secondary goat anti‐rabbit Alexa 568 antibody was obtained from Invitrogen. The BACE inhibitor (β‐secretase inhibitor IV, CAS 797035‐11‐1) was purchased from Merck Millipore. Galardin (GM6001) was obtained from Biomol, and Dyngo 4A was obtained from Abcam. Transferrin‐Alexa 594 was a kind gift from Felipe Opazo, and MβCD was purchased from Sigma‐Aldrich.

### Aβ‐peptide measurement

The concentrations of Aβ‐proteins (Aβ_1–42_, Aβ_1–40_, Aβ_x–42_ and Aβ_x–40_) in the samples were measured via the INNO‐BIA plasma Aβ forms multiplexing assay (Innogenetics) as described previously 24 with the exception that the samples in the current study were diluted 1:2 rather than 1:3. Briefly, in this multiplexing assay, the beads were coated with C‐terminal‐specific antibodies that bound to Aβ_42_ or Aβ_40_ as appropriate. A non‐Aβ‐binding antibody was added to account for plasma matrix interference, such as that from heterophilic antibodies. For detection, a second monoclonal antibody was used that was either specific for residue 1 of the N‐terminus (Module A) or was nonspecific (Module B); that is, Module B also recognized N‐terminally modified (i.e. shortened) Aβ peptides. The resulting fluorescence signals were detected using a Luminex 100 IS analyzer (Luminex). All analyses were performed in duplicate.

### Western blot

HeLa cells were cultured in six‐well plates. After 24 h, the cells were transfected with pHAPP or pHBACE or were untransfected as a control. On day 3, the media were collected, after which the cells were lysed using 300 μL per well of RIPA buffer (45 mm Tris–HCl, 150 mm NaCl, 1% NP‐40, 0.5% sodium deoxycholate and 0.5% SDS) containing a protease inhibitor (cOmplete, EDTA‐free Protease Inhibitor Cocktail Tablets, Roche Diagnostics). The protein lysate and media samples were denatured, and 20 μL of the lysate or media was then loaded in each well of SDS–PAGE gels (4–15% Mini‐PROTEAN^®^ TGX™ Precast Gel, Bio‐Rad). Under semi‐dry conditions, the proteins were electrophoretically transferred to a nitrocellulose membrane, which was then blocked in 2.5% milk in PBST buffer (PBS, Biochrom; containing 1% Tween‐20) for 2 h at room temperature. Subsequently, the membranes were washed three times with PBST (with gentle shaking). The primary anti‐GFP antibody (1:2000) diluted in 0.5% milk in PBST buffer was then applied to the membrane, followed by incubation overnight at 4°C. An antibody against GAPDH was used (1:20 000) as a loading control. Following another PBST washing step, the membrane was incubated in the horseradish peroxidase‐labeled secondary antibody (diluted 1:20 000 in 0.5% milk in PBST buffer) for 2 h at room temperature, washed again in PBST buffer and then treated with ECL Prime solution (Bio‐Rad). The luminescence signal was detected using the Fluor‐S^®^ MAX MultiImaging System (Bio‐Rad). The MagicMark™ XP Western Protein Standard (20–220, Life Technologies) was used as the MW marker. Analysis was performed using matlab (MathWorks,) software; specifically, known masses of the MW markers and the local maxima of their corresponding bands were used to generate an MW curve. The exponential fit of the MW curve was then used to calculate the exact molecular mass of the sample bands via interpolation.

### Imaging

The experiments were conducted at room temperature using a Nikon TI‐Eclipse inverted microscope (Nikon [Tokio, Japan]) equipped with a 100×, 1.49 NA oil immersion objective (or a 60×, 1.2 NA water immersion objective for epifluorescence microscopy) using the Perfect Focus System™ (Nikon [Tokio, Japan]). The fluorophores were excited using a Nikon D‐Eclipse C1 laser box at wavelengths of 488 and 561 nm for TIRF microscopy or using a Nikon Intensilight C‐HGFI for epifluorescence microscopy. For single‐color imaging, excitation filters of 482/18 and 561/14 nm employing dichroic long‐pass mirrors (threshold wavelengths of 488 and 561 nm, respectively) were used. The emitted light was passed through emission bandpass filters of 525/45 and 609/54 nm. For dual‐color imaging, dual‐band excitation filters with ranges of 455–490 nm and 560–590 nm were used with a dichroic long‐pass mirror (threshold wavelength of 560 nm). No emission filters were used. Instead, an image splitter containing a dichroic long‐pass mirror (threshold wavelength of 535 nm) and two emission bandpass filters of 520/35 and 605/70 nm were attached to the microscope. All filters were purchased from Semrock. Finally, the emitted light was projected onto a cooled EM‐CCD camera (iXon^EM^ DU‐885 or DU‐897, Andor).

The coverslips were placed in a perfusion chamber (volume = 500 μL) containing saline (144 mm NaCl, 2.5 mm KCl, 2.5 mm CaCl_2_, 2.5 mm MgCl_2_, 10 mm glucose and 10 mm HEPES, pH 7.5).

For the acidic and NH_4_^+^‐containing solution experiments, we employed a three‐channel perfusion system using a pH 7.5 buffer, a pH 5.5 buffer and a pH 7.5 buffer containing 50 mm NH_4_Cl. Rapid solution exchanges were performed using a piezo‐controlled stepper device (SF‐77B; Warner Instruments) and three‐barrel glass tubing. The results of the experiments were recorded using Andor Solis (Andor) or Nis Elements (Nikon) software.

### Immunocytochemistry

For immunocytochemistry, HeLa cells were cultured directly on glass coverslips using standard culture techniques. All labeling steps were performed at room temperature. The culture medium was removed, and the cells were washed in Dulbecco's PBS (Invitrogen) prior to fixation in 4% Roti‐Histofix (Carl Roth) for 5 min. The Roti‐Histofix was then removed by washing the cells with PBS prior to permeabilization using 0.1% Triton X‐100 in Roti‐Immunoblock (Carl Roth). Next, the cells were washed and blocked for 45 min using Roti‐Immunoblock. Primary antibodies against APP and BACE were then added to the Roti‐Immunoblock, followed by incubation for 16 h. After washing, the cells were incubated in secondary antibodies (goat anti‐mouse Alexa 568 and goat anti‐rabbit Alexa 568) diluted in Roti‐Immunoblock for 1 h at room temperature. The coverslips were then washed again three times prior to mounting and were subjected to immunofluorescence analysis.

### Image analysis

To investigate the exocytosis kinetics, the images were viewed using Nis Elements software (Nikon), and fusion events were detected manually. To evaluate the frequency of fusion events, the image stacks were used to automatically define an ROI for each fusion event 49. The resulting fluorescence profiles were then manually inspected for putative fusion events.

Statistical analyses were performed using matlab software. The error bars indicate the standard error of the mean (SEM) unless otherwise indicated.

## Supporting information


**Figure S1: Endocytosis is blocked in HeLa cells using Dyngo 4A.** Untransfected HeLa cells were incubated in 10 µm Dyngo 4A or 0.1% DMSO as a control. After 30 min, 25 µg/mL transferrin‐Alexa 594 was added for 20 min. Subsequently, the cells were washed and imaged in Dyngo 4A‐ or DMSO‐containing Tyrode's solution via epifluorescence microscopy. While transferrin‐Alexa 594 was affiliated with and enriched in the DMSO‐treated cells [mean intensity = 1028.38 AU (±66.26 AU)], there was only minimal transferrin‐Alexa 594 detected on the surfaces of Dyngo 4A‐treated cells [mean intensity = 642.33 AU (±56.04 AU)] (p_TTEST_ = 9.35 × 10^−5^; N = 3).

**Figure S2: Exocytosis of mOrBACE displays the same kinetic properties as pHBACE.** A) Normalized average fluorescence profile of the mOrBACE fusion events (n > 15). For comparison, the normalized average fluorescence profile of pHBACE from Figure 2A is shown. pHBACE and mOrBACE displayed the same kinetics [τ_pHBACE_ = 2.28 seconds (±0.48), τ_mOrBACE_ = 2.08 seconds (±0.74)]. B) MSD for pHAPP (green dots) and mOrBACE (red dots). The slope of the linear fits (gray line) corresponds to the diffusion coefficient (D_mOrBACE_ = 0.27 µm^2^/second, n > 10), which is in the same range as that of pHBACE (D_pHrBACE_ = 0.47 µm^2^/second, n > 20).

**Movie S1:** pHAPP. HeLa cells transfected with pHAPP were imaged via TIRF microscopy (Nikon TI‐Eclipse inverted microscope). Frames were captured at 5 Hz for 1 min.

**Movie S2.** pHBACE. HeLa cells transfected with pHBACE were imaged via TIRF microscopy (Nikon TI‐Eclipse inverted microscope). Frames were captured at 5 Hz for 1 min.
