# Supplementary material for: Surface Trafficking of APP and BACE in Live Cells
Source: Traffic. 2015 Apr 14;16(6):655–75. doi: 10.1111/tra.12270 (PMC6680167; doi:10.1111/tra.12270)
Supplement: Supplementary file 1 — Figure S1: Endocytosis is blocked in HeLa cells using Dyngo 4A. Untransfected HeLa cells were incubated in 10 µm Dyngo 4A or 0.1% DMSO as a control. After 30 min, 25 µg/mL transferrin‐Alexa 594 was added for 20 min. Subsequently, the cells were washed and imaged in Dyngo 4A‐ or DMSO‐containing Tyrode's solution via epifluorescence microscopy. While transferrin‐Alexa 594 was affiliated with and enriched in the DMSO‐treated cells [mean intensity = 1028.38 AU (±66.26 AU)], there was only minimal transferrin‐Alexa 594 detected on the surfaces of Dyngo 4A‐treated cells [mean intensity = 642.33 AU (±56.04 AU)] (p TTEST = 9.35 × 10^−5^; N = 3). [file TRA-16-655-s001.doc]

**
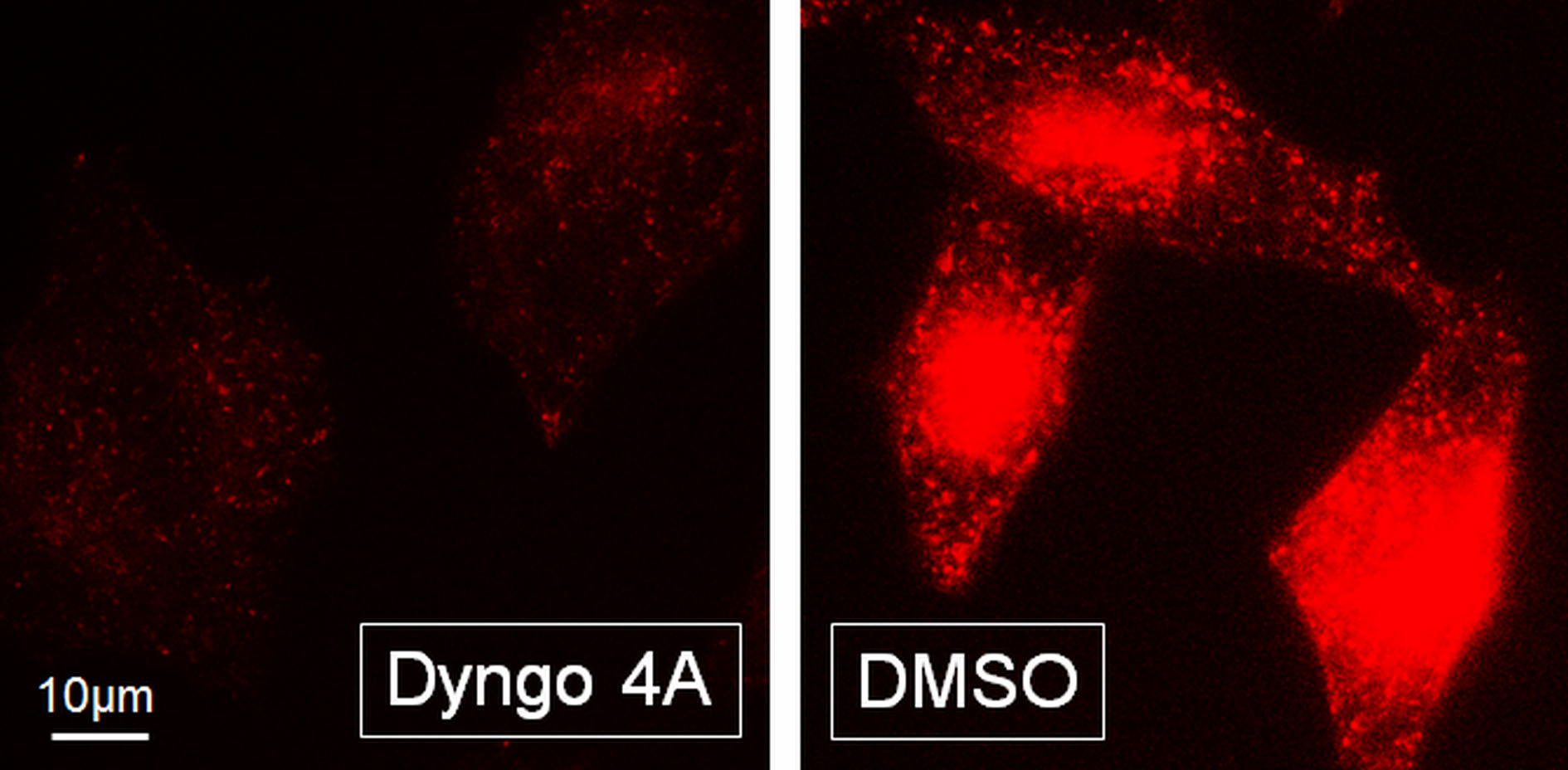
**

**Figure S1. Endocytosis is blocked in HeLa cells using Dyngo 4A.**

Untransfected HeLa cells were incubated in 10 µM Dyngo 4A or 0.1% DMSO as a control. After 30 minutes, 25 µg/ml Transferrin-Alexa 594 was added for 20 minutes. Subsequently, the cells were washed and imaged in Dyngo 4A- or DMSO-containing Tyrode’s solution via epifluorescence microscopy. Whereas Transferrin-Alexa 594 was affiliated with and enriched in the DMSO-treated cells (mean intensity=1028.38 AU (+/-66.26 AU)), there was only minimal Transferrin-Alexa 594 detected on the surfaces of Dyngo 4A-treated cells (mean intensity=642.33 AU (+/-56.04 AU)) (*p_TTEST_=9.35*10^-5^; N=3)*.
