# Supplementary material for: Surface Trafficking of APP and BACE in Live Cells
Source: Traffic. 2015 Apr 14;16(6):655–75. doi: 10.1111/tra.12270 (PMC6680167; doi:10.1111/tra.12270)
Supplement: Supplementary file 2 — Figure S2: Exocytosis of mOrBACE displays the same kinetic properties as pHBACE. A) Normalized average fluorescence profile of the mOrBACE fusion events (n > 15). For comparison, the normalized average fluorescence profile of pHBACE from Figure 2A is shown. pHBACE and mOrBACE displayed the same kinetics [τ pHBACE = 2.28 seconds (±0.48), τ mOrBACE = 2.08 seconds (±0.74)]. B) MSD for pHAPP (green dots) and mOrBACE (red dots). The slope of the linear fits (gray line) corresponds to the diffusion coefficient (D mOrBACE = 0.27 µm2/second, n > 10), which is in the same range as that of pHBACE (D pHrBACE = 0.47 µm2/second, n > 20). [file TRA-16-655-s002.doc]

**
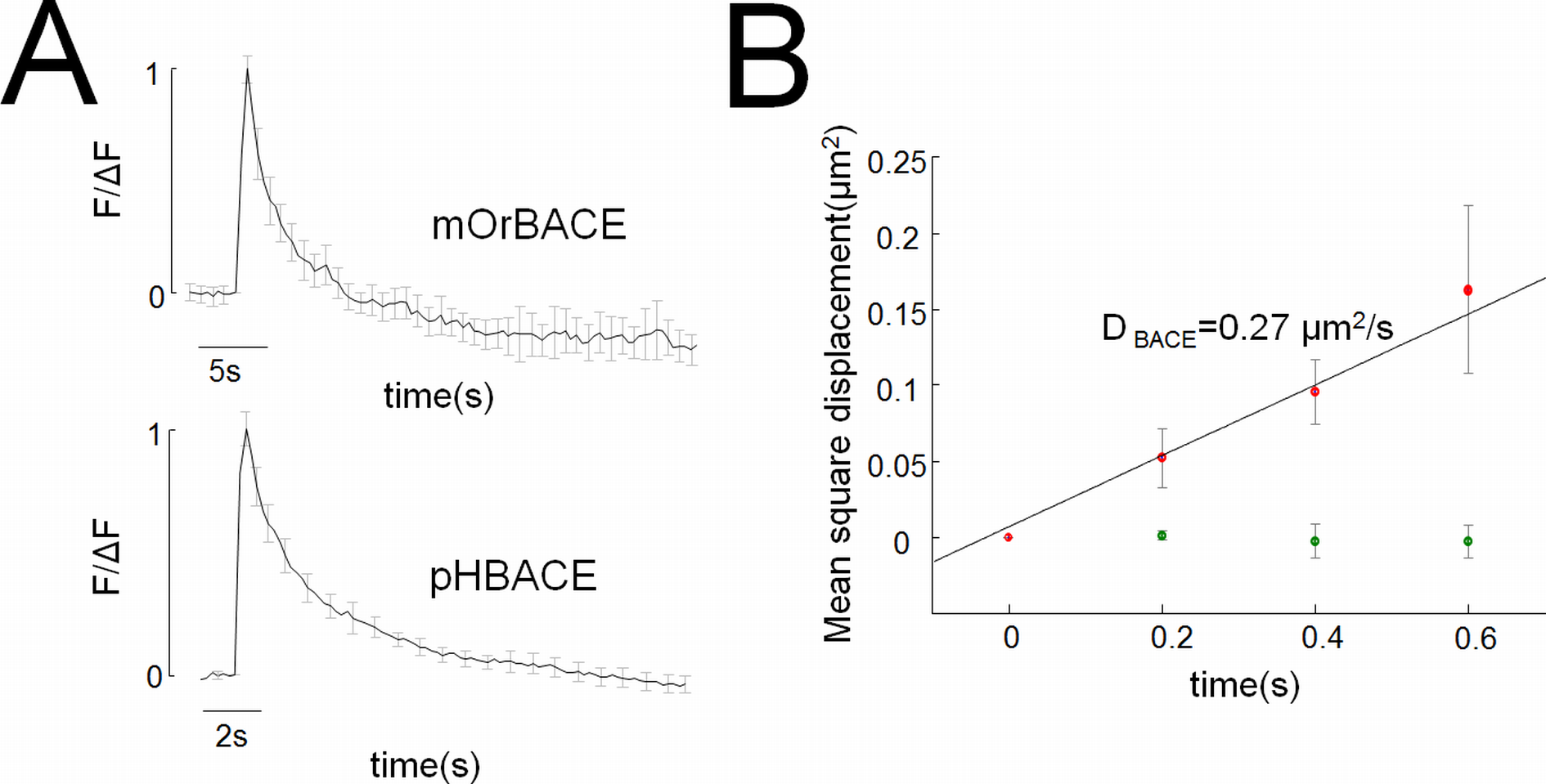
**

**Figure S2. Exocytosis of mOrBACE displays the same kinetic properties as pHBACE.**

(A) Normalized average fluorescence profile of the mOrBACE fusion events (n>15). For comparison, the normalized average fluorescence profile of pHBACE from Fig. 2A is shown. pHBACE and mOrBACE displayed the same kinetics (τ_pHBACE_=2.28 s (+/-0.48), τ_mOrBACE_=2.08 s (+/-0.74). (B) MSD for pHAPP (green dots) and mOrBACE (red dots). The slope of the linear fits (grey line) corresponds to the diffusion coefficient (D_mOrBACE_=0.27 µm2/s, n>10), which is in the same range as that of pHBACE (D_pHrBACE_=0.47 µm2/s, n>20).
